# Focal ischemic stroke leads to lung injury and reduces alveolar macrophage phagocytic capability in rats

**DOI:** 10.1186/s13054-018-2164-0

**Published:** 2018-10-05

**Authors:** Cynthia S. Samary, Alane B. Ramos, Lígia A. Maia, Nazareth N. Rocha, Cíntia L. Santos, Raquel F. Magalhães, Amanda L. Clevelario, Pedro M. Pimentel-Coelho, Rosália Mendez-Otero, Fernanda F. Cruz, Vera L. Capelozzi, Tatiana P. T. Ferreira, Thea Koch, Marcelo Gama de Abreu, Claudia C. dos Santos, Paolo Pelosi, Pedro L. Silva, Patricia R. M. Rocco

**Affiliations:** 1 Laboratory of Pulmonary Investigation, Carlos Chagas Filho Biophysics Institute, Federal University of Rio de Janeiro, Centro de Ciências da Saúde, Avenida Carlos Chagas Filho, s/n, Bloco G-014, Ilha do Fundão, Rio de Janeiro, RJ 21941-902 Brazil; 2 Laboratory of Cellular and Molecular Neurobiology, Carlos Chagas Filho Biophysics Institute, Federal University of Rio de Janeiro, Rio de Janeiro, RJ Brazil; 3 Department of Physiology and Pharmacology, Fluminense Federal University, Niteroi, RJ Brazil; 4 Department of Pathology, School of Medicine, University of São Paulo, São Paulo, SP Brazil; 5 Laboratory of Inflammation, Oswaldo Cruz Institute, Oswaldo Cruz Foundation (FIOCRUZ), Rio de Janeiro, RJ Brazil; 6 Pulmonary Engineering Group, Department of Anesthesiology and Intensive Care Therapy, University Hospital Carl Gustav Carus, Technische Universität Dresden, Dresden, Germany; 7 Interdepartmental Division of Critical Care, Keenan Research Centre for Biomedical Science of St. Michael’s Hospital, University of Toronto, Toronto, ON Canada; 8 Dipartimento di Scienze Chirurgiche e Diagnostiche Integrate (DISC), Università degli Studi di Genova, Genoa, Italy; 9 IRCCS Ospedale Policlinico San Martino, Genoa, Italy

**Keywords:** Focal ischemic stroke, Lung injury, Brain–lung interaction, Inflammation, Macrophages

## Abstract

**Background:**

Ischemic stroke causes brain inflammation, which we postulate may result in lung damage. Several studies have focused on stroke-induced immunosuppression and lung infection; however, the possibility that strokes may trigger lung inflammation has been overlooked. We hypothesized that even focal ischemic stroke might induce acute systemic and pulmonary inflammation, thus altering respiratory parameters, lung tissue integrity, and alveolar macrophage behavior.

**Methods:**

Forty-eight Wistar rats were randomly assigned to ischemic stroke (Stroke) or sham surgery (Sham). Lung function, histology, and inflammation in the lung, brain, bronchoalveolar lavage fluid (BALF), and circulating plasma were evaluated at 24 h. In vitro, alveolar macrophages from naïve rats (unstimulated) were exposed to serum or BALF from Sham or Stroke animals to elucidate possible mechanisms underlying alterations in alveolar macrophage phagocytic capability. Alveolar macrophages and epithelial and endothelial cells of Sham and Stroke animals were also isolated for evaluation of mRNA expression of interleukin (IL)-6 and tumor necrosis factor (TNF)-α.

**Results:**

Twenty-four hours following ischemic stroke, the tidal volume, expiratory time, and mean inspiratory flow were increased. Compared to Sham animals, the respiratory rate and duty cycle during spontaneous breathing were reduced, but this did not affect lung mechanics during mechanical ventilation. Lungs from Stroke animals showed clear evidence of increased diffuse alveolar damage, pulmonary edema, and inflammation markers. This was associated with an increase in ultrastructural damage, as evidenced by injury to type 2 pneumocytes and endothelial cells, cellular infiltration, and enlarged basement membrane thickness. Protein levels of proinflammatory mediators were documented in the lung, brain, and plasma (TNF-α and IL-6) and in BALF (TNF-α). The phagocytic ability of macrophages was significantly reduced. Unstimulated macrophages isolated from naïve rats only upregulated expression of TNF-α and IL-6 following exposure to serum from Stroke rats. Exposure to BALF from Stroke or Sham animals did not change alveolar macrophage behavior, or gene expression of TNF-α and IL-6. IL-6 expression was increased in macrophages and endothelial cells from Stroke animals.

**Conclusions:**

In rats, focal ischemic stroke is associated with brain–lung crosstalk, leading to increased pulmonary damage and inflammation, as well as reduced alveolar macrophage phagocytic capability, which seems to be promoted by systemic inflammation.

**Electronic supplementary material:**

The online version of this article (10.1186/s13054-018-2164-0) contains supplementary material, which is available to authorized users.

## Background

Ischemic stroke is the second leading cause of death worldwide and will affect at least one-sixth of the population [1], with significant morbidity and mortality [2]. Stroke patients require intensive care unit admission for close monitoring [2]. Survivors experience long-term disability and significantly impaired quality of life [3].

Ischemic stroke leads to cerebral cell death, causing brain inflammation and neurological deficits [4]. In addition to local immunoinflammatory responses in the brain, stroke also triggers systemic responses, which may have important distal organ effects.

Notably, the lungs are particularly vulnerable in the event of severe brain damage, including ischemic and hemorrhagic strokes [5–8]. Bai et al. [7] reported that 15.6% of stroke patients developed acute lung injury within 36 h of hospital admission, and 7.8% of patients had pneumonia or bronchitis during hospitalization. Studies in experimental models of stroke have focused on stroke-induced immunosuppression and lung infection [9–13], whereas lung injury and inflammation, and their effects on the phagocytic capability of alveolar macrophages, have been overlooked. This is in contrast to the literature on traumatic brain injury, where lung inflammation has been well characterized in a variety of animal models [14–18].

We hypothesized that stroke might induce acute systemic and lung inflammation affecting respiratory parameters, pulmonary histological features, and alveolar macrophage phagocytic behavior. In this context, we investigated the impact of focal ischemic stroke on the respiratory pattern, lung histology, behavior of innate immune cells that reside in the lung, and activation of the innate immune system in the brain, lung, and circulatory compartments. Additionally, complementary in-vitro studies were conducted to elucidate the possible mechanisms underlying changes in the behavior of professional phagocytes, alveolar macrophages, following stimulation with bronchoalveolar lavage fluid (BALF) or serum isolated from naïve (unstimulated) or stroke rats. The expression of proinflammatory mediators was also evaluated in alveolar macrophages and epithelial and endothelial cells isolated from Sham and Stroke animals.

## Methods

### Study approval

This study was approved by the Animal Care Committee of the Health Sciences Center, Federal University of Rio de Janeiro (CEUA: 145/13). Detailed methods are described in Additional file 1.

### Animal preparation and experimental protocol

Forty-eight male Wistar rats (weight 350–400 g) were anesthetized (xylazine 2.5 mg/kg intraperitoneally (i.p.) and ketamine 75 mg/kg i.p.) and then randomly allocated to undergo ischemic stroke induction by thermocoagulation of pial vessels over the right primary sensorimotor cortex (Stroke) or sham surgery (Sham). After 24 h, 12 animals underwent the cylinder test for analysis of forelimb use asymmetry and brain magnetic resonance imaging (MRI) to confirm the presence of cortical ischemic stroke. Animals were then placed separately in closed chambers for noninvasive plethysmography [19]. At the end of the experiment, the lungs were removed for histology. In 12 other rats, lung mechanics were evaluated invasively and the BALF, blood, lungs, and brains were harvested for molecular biology analyses. In 12 additional naïve Wistar rats, alveolar macrophages were extracted and incubated with either serum or BALF collected from Sham or Stroke rats to evaluate phagocytic capability and mRNA expression of interleukin (IL)-6 and tumor necrosis factor (TNF)-α. Finally, 12 additional rats (*n* = 6/group) were used to evaluate protein levels of IL-6 and TNF-α in lung and brain tissue homogenates. Alveolar macrophages, epithelial cells, and endothelial cells were also isolated from Sham and Stroke animals to analyze mRNA expression of IL-6 and TNF-α in these cells.

At the end of all experiments, animals were euthanized by sodium thiopental overdose (150 mg/kg i.p.).

### Carotid Doppler ultrasonography

Carotid Doppler ultrasonography was performed under anesthesia (xylazine 2.5 mg/kg intraperitoneally (i.p.) and ketamine 75 mg/kg i.p.) before and after focal ischemic stroke. The peak systolic velocity (PSV), diastolic velocity (DV), and resistive index ($PSV-\frac{DV}{PSV}$) were measured [20].

### Surgery

Anesthetized rats were placed in a stereotactic frame and their heads immobilized. Ischemia was induced by thermocoagulation of pial blood vessels overlying the primary somatosensory, motor, and sensorimotor cortices, as described elsewhere [21]. Sham surgery consisted of the same procedure without thermocoagulation.

### Cylinder test

At 24 h, animals underwent forelimb use asymmetry testing [22]. For each animal, the percentage relative to the total number of uses (ipsilateral + contralateral + simultaneous) was calculated for ipsilateral (unimpaired) and contralateral (impaired) uses. An asymmetry score was then calculated for each animal (% of ipsilateral uses − % of contralateral uses). The resulting score was then converted to a symmetry score (100 − asymmetry score).

### Magnetic resonance imaging

At 24 h, MRI was performed in anesthetized rats. Images were acquired in a 7 T scanner (7 T/210 Horizontal Varian; Agilent Technologies, Palo Alto, CA, USA), using fast spin echo (FSE) proton density (PD) sequences (matrix 192 × 192; slice thickness 0.5 mm; 15 continuous slices) in the axial (TR/TE 1500/11 ms; FOV 30 × 30 cm^2^), coronal (TR/TE 2100/11 ms; FOV 30 × 30.5 cm^2^), and sagittal (TR/TE 1500/11 ms; FOV 30 × 30.5 cm^2^) planes. Data were processed in VnmrJ software (Agilent) and an observational analysis was performed.

### Ventilatory parameters

Conscious, spontaneously breathing rats were placed in a whole-body plethysmography system (FinePointe™ R/C Buxco; Buxco Eletronics, Sharon, CT, USA). The tidal volume (V_T_), respiratory rate (RR), inspiratory time (T_I_), expiratory time (T_E_), total (T_TOT_) time, mean inspiratory flow (V_T_/T_I_), and duty cycle (T_I_/T_TOT_) were measured [23].

### Invasive lung mechanics assessment

Animals were premedicated i.p. with diazepam (10 mg/kg), ketamine (75 mg/kg), and xylazine (2.5 mg/kg). Tracheotomy was performed, and neuromuscular blockade was achieved with vecuronium bromide (2 mg/kg i.v.). Animals were mechanically ventilated (Servo-I; MAQUET, Solna, Sweden) in volume-controlled mode, with V_T_ = 6 ml/kg, RR = 80 breaths/min, FiO_2_ = 0.4, and positive end-expiratory pressure (PEEP) = 3 cmH_2_O for 5 min. Airflow and airway and esophageal pressures were continuously recorded. Airway resistance and static lung elastance were computed by the end-inflation occlusion method [24].

### Bronchoalveolar lavage fluid

After lung removal at PEEP = 3 cmH_2_O, the left bronchus was cannulated. Phosphate-buffered saline solution was instilled (1.5 ml, 37 °C) and aspirated three times. Samples were centrifuged at 1500 × *g* for 10 min at 4 °C, and then stored at − 80 °C for further analysis. Protein content was determined by the Bradford method [25].

### Phagocytic capability of alveolar macrophages

The phagocytic capability of alveolar macrophages was tested with pH-sensitive pHrodo™ Green Zymosan A BioParticle® (Life Technologies, Carlsbad, CA, USA) conjugate for phagocytosis, as per the supplier’s instructions. pHrodo™ Green conjugates are nonfluorescent outside the cell at neutral pH, but fluoresce bright green at acidic pH, such as in phagosomes. Cells collected from BALF were seeded on a tissue culture dish and incubated in RPMI 1640 10% fetal bovine serum (FBS) with 1% penicillin/streptomycin for 2 h at 37 °C, 5% CO_2._ Cells that adhere to the plate are macrophages [26]*.* Briefly, a total of 10^5^ alveolar macrophages were plated on a 96-well plate. Cells were washed with saline (0.9% NaCl) and incubated with fluorescently pHrodo™ Green-labeled *S. cerevisiae* particles (0.5 mg/ml) for 2 h. After incubation, the cells were placed on ice to halt phagocytosis, washed twice with ice-cold PBS, and prepared for analysis. Fluorescence was measured in a microplate reader (Perkin-Elmer, Waltham, MA, USA). Phagocytosis of fluorescently labeled BioParticles® was quantified by measuring intracellular fluorescence emitted by engulfed particles at 585 nm.

### Lung histology

The right lung was fixed in 4% buffered formalin and embedded in paraffin. Sections (3 μm thick) were stained with hematoxylin and eosin. Photomicrographs at magnifications of × 25, × 100, and × 400 were obtained from eight nonoverlapping fields of view per section using a light microscope (Olympus BX51; Olympus Latin America Inc., Brazil). Diffuse alveolar damage (DAD) and bronchoconstriction were quantified using a weighted scoring system, as previously described [27].

### Transmission electron microscopy

Three slices (2 mm × 2 mm × 2 mm) were cut from three different segments of the left lung for electron microscopy. On each micrograph (20 fields per animal), damage to the alveolar capillary membrane, type 2 epithelial and endothelial cells, basement membrane thickness, macrophages, and degree of interstitial edema were graded on a 5-point, semiquantitative, severity-based scoring system as described elsewhere (see Additional file 1) [24].

### Real-time PCR

Quantitative real-time reverse transcription polymerase chain reaction (RT-PCR) was performed to measure biological markers associated with inflammation (IL-6 and TNF-α) and the housekeeping gene *36B4* in peri-lesion slices of brain, central slices of left lung, as well as isolated alveolar macrophages and epithelial and endothelial cells. The primer sequences are presented in Additional file 2: Table S1. For each sample measured in triplicate, gene expression was normalized to that of *36B4* and expressed as the fold-change relative to Sham animals, using the 2^− ΔΔ^Ct method, where ΔCt = Ct (target gene) – Ct (reference gene). This is a suitable method to analyze relative changes in gene expression from quantitative real-time PCR experiments [28].

### Enzyme-linked immunosorbent assay

IL-6 and TNF-α levels were quantified in lung and brain tissue homogenates, BALF, and plasma by enzyme-linked immunosorbent assay (ELISA) following the manufacturer’s recommendations [29].

### In-vitro experiments

Alveolar macrophages from naïve Wistar rats were extracted and then incubated with either serum or BALF collected from Sham or Stroke rats (24 h after stroke or the sham procedure) for 24 h. Phagocytic capability was analyzed as already described. RNA from these cells was extracted and further evaluated by quantitative RT-PCR for the TNF-α and IL-6 genes.

Alveolar macrophages and epithelial and endothelial cells were extracted from Sham and Stroke rats. Gene expression of TNF-α and IL-6 was evaluated by quantitative RT-PCR.

### Statistical analysis

Sample size calculation was based on effect estimates obtained from previous studies in rodents using similar settings [30]. The Student *t* test and the Mann–Whitney *U* test were used for parametric and nonparametric data, respectively. Parametric data are expressed as mean ± SD, and nonparametric data as median (interquartile range). All tests were performed using GraphPad Prism v6.07 (GraphPad Software, La Jolla, CA, USA). Significance was established at *p* < 0.05.

## Results

### Focal ischemic stroke model

Focal ischemic stroke was confirmed by forelimb motor asymmetry (Additional file 3: Figure S1). Additional file 4: Figure S2 illustrates cerebral infarction in a representative animal (MRI). Peak systolic velocity ipsilateral to brain damage decreased in rats after stroke (right carotid, 52 ± 33 cm/s), while no significant changes were observed in the contralateral side (left carotid, 64 ± 32 cm/s). The resistive index did not differ between groups (Additional file 5: Figure S3).

### Lung function analysis

**Fig. 1:**
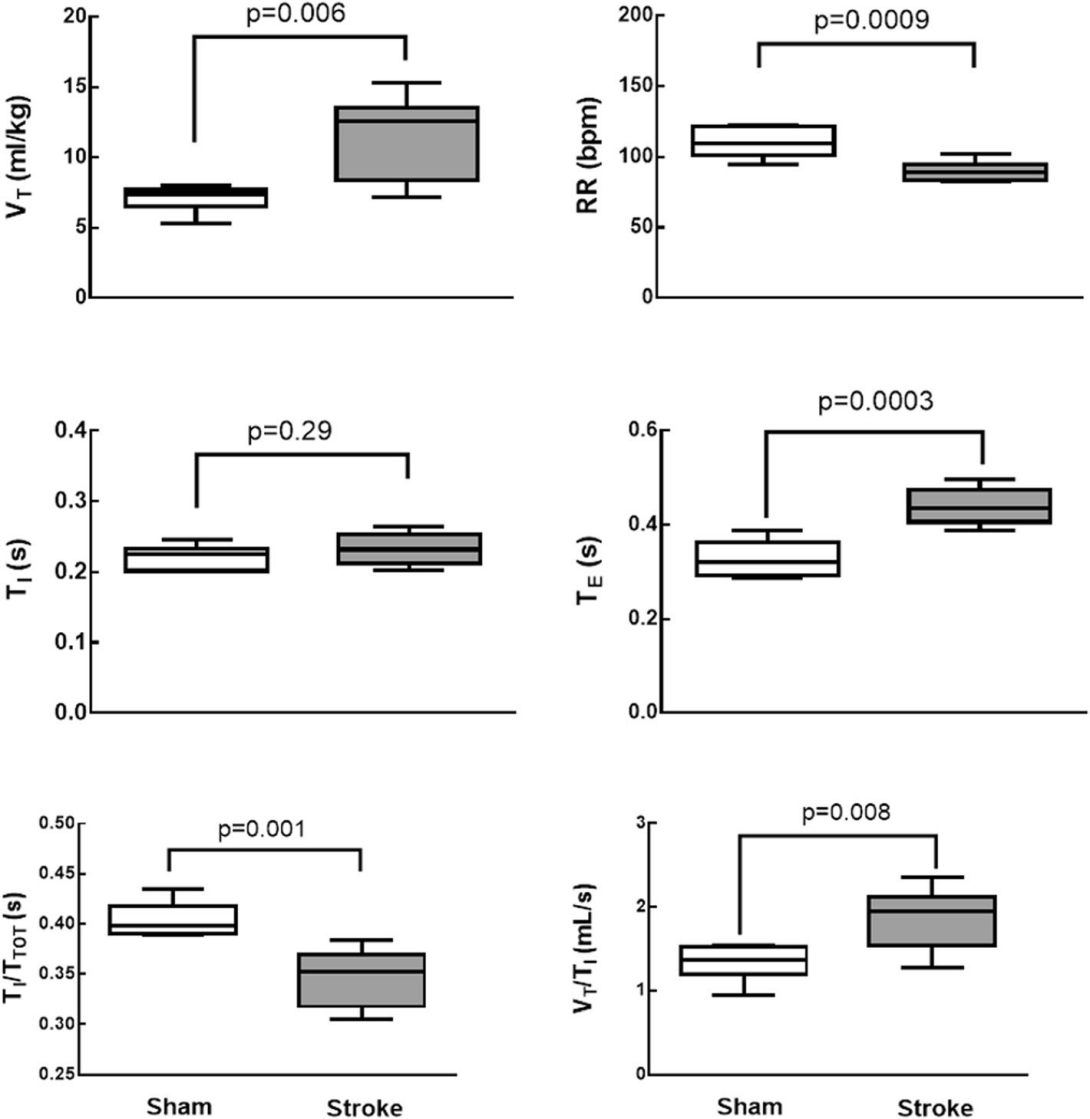
Plethysmography analysis in Sham and ischemic stroke (Stroke) groups. Boxes show interquartile (25–75%) range, whiskers denote range (minimum–maximum), horizontal lines represent median in 6 animals/group. V_T_ tidal volume, RR respiratory rate, T_I_ inspiratory time, T_E_ expiratory time, T_I_/T_TOT_ duty cycle, V_T_/T_I_ mean inspiratory flow

Compared to sham animals, V_T_, T_E_, and the V_T_/T_I_ ratio were increased in the stroke group, while RR and TI/T_TOT_ were reduced (Fig. 1). Lung mechanics (Additional file 6: Table S2) and arterial blood gases (Additional file 7: Table S3) did not differ significantly between groups**.**

### Histological and ultrastructural features

DAD was higher in Stroke animals compared to Sham animals (Table 1) due to increased edema and inflammation. Atelectasis did not differ significantly between the Stroke and Sham groups. Bronchoconstriction was observed only in Stroke animals (Table 1; Additional file 8: Figure S4).

**Table 1:** Diffuse alveolar damage score

Parameter	Sham group	Stroke group
Lung parenchyma		
Edema (0–16)	1 (0.25–1.75)	6 (6.0–8.25)*
Inflammation (0–16)	0.5 (0.0–1.75)	2.5 (2.0–3.75)**
Atelectasis (0–16)	2.0 (2.0–3.5)	4.5 (3.0–6.0)
Cumulative DAD score (0–48)	4.0 (4.0–4.75)	14 (12.5–15.5)**
Airways		
Bronchoconstriction (0–16)	1.0 (1.0–1.0)	12 (10.5–12)***

Cumulative diffuse alveolar damage (DAD) score representing injury from interstitial edema, inflammation, and atelectasis, as well as bronchoconstriction in Sham and Stroke groups. Values are median (interquartile range) of 6 animals/group

^*^p < 0.001

^**^p < 0.0001

^***^p < 0.05 vs Sham

Transmission electron microscopy showed intra-alveolar edema and increased macrophage counts, epithelial and endothelial cell damage, basement membrane thickness, and collagen fiber content in Stroke animals (Additional file 9: Figure S5; Additional file 10: Table S4).

### Inflammation and macrophage phagocytic capability

**Fig. 2:**
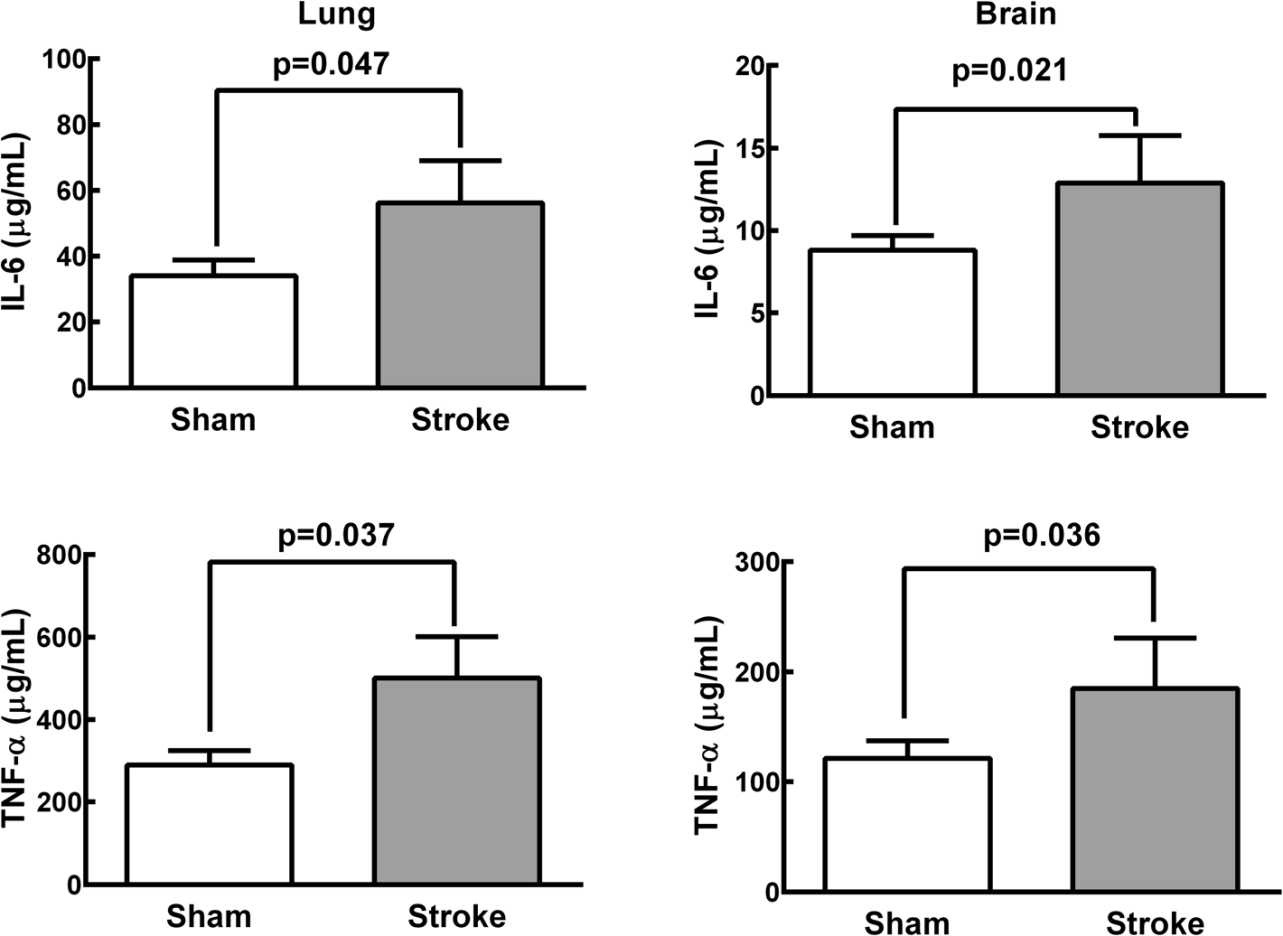
Protein levels of interleukin (IL)-6 and tumor necrosis factor (TNF)-α in lung and brain tissue homogenates in Sham and Stroke groups. Values are mean ± SD of 6 animals/group

Stroke rats exhibited significantly higher gene expression (Additional file 11: Figure S6) and protein levels (Fig. 2) of TNF-α and IL-6 in brain and lung tissue homogenates compared to Sham animals. Ischemic stroke significantly increased protein levels of TNF-α in BALF and plasma, as well as of IL-6 in plasma (Additional file 12: Figure S7). Total protein levels in BALF were higher in Stroke animals than in Sham animals (Additional file 13: Figure S8).

**Fig. 3:**
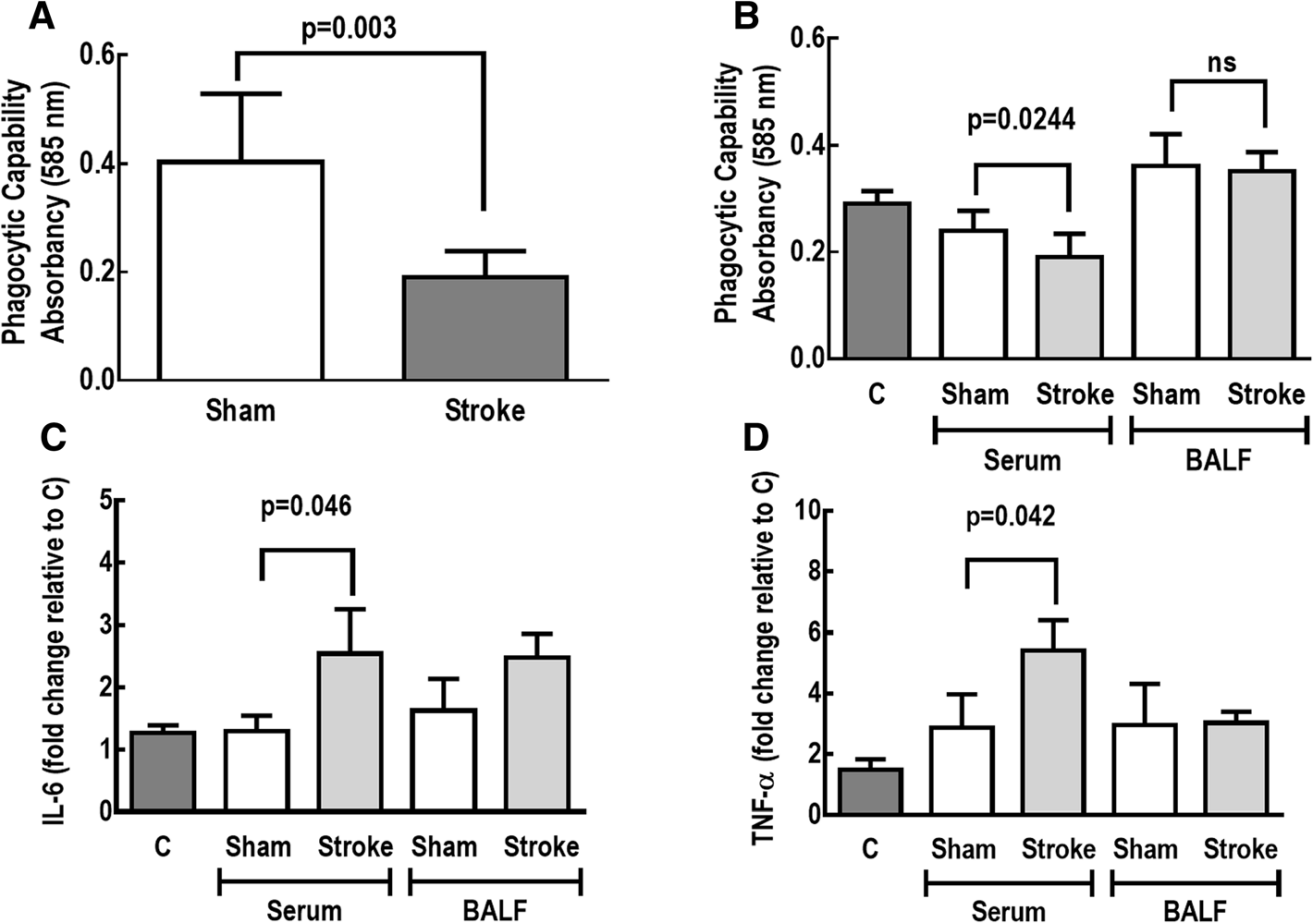
Phagocytic capability of alveolar macrophages in Sham and Stroke animals (**a**). Alveolar macrophages were exposed to serum and bronchoalveolar lavage fluid (BALF) from Sham and Stroke animals (*n* = 6/each). Phagocytic capability (**b**) as well as gene expressions of interleukin (IL)-6 (**c**) and tumor necrosis factor (TNF)-α (**d**) were analyzed. Values are mean ± SD of 6 animals/group. C unstimulated alveolar macrophages

The phagocytic capability of alveolar macrophages was decreased in Stroke animals (Fig. 3). In addition, the phagocytic behavior of alveolar macrophages obtained from naïve animals was reduced, whereas gene expression of TNF-α and IL-6 in these cells was increased, after exposure to serum from Stroke (but not Sham) rats. In contrast, exposure to BALF from Stroke or Sham animals did not change the phagocytic capability of alveolar macrophages, or the gene expression of IL-6 and TNF-α (Fig. 3).

**Fig. 4:**
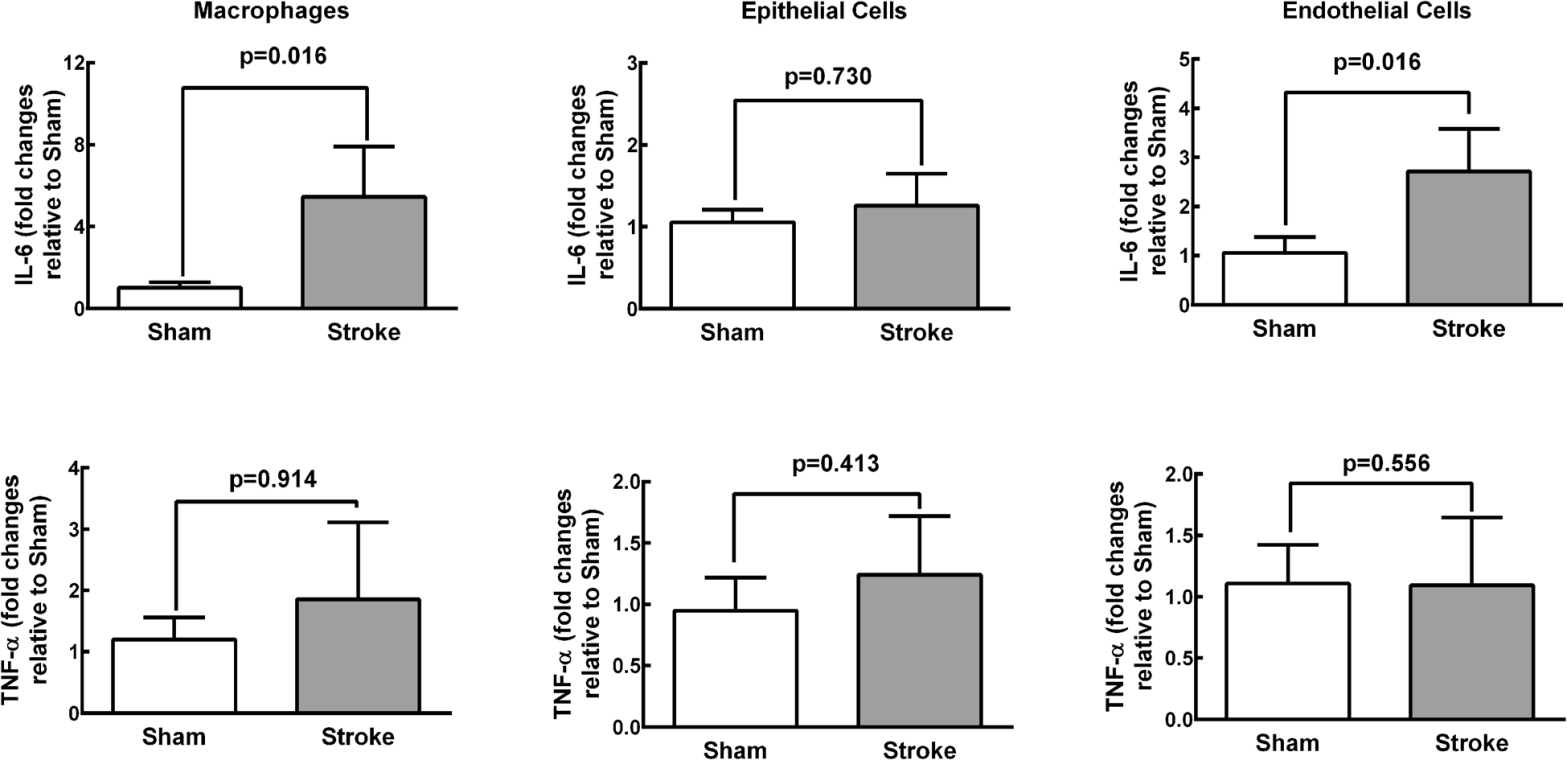
Real-time PCR analysis of biological markers associated with inflammation (interleukin (IL)-6 and tumor necrosis factor (TNF)-α) in alveolar macrophages and epithelial and endothelial cells isolated from Sham and Stroke groups. Values are mean ± SD of 4–6 animals/group

Gene expression of IL-6 was higher in alveolar macrophages and endothelial cells from Stroke animals compared to Sham animals. No significant differences were observed in IL-6 expression in epithelial cells or TNF-α expression in alveolar macrophages, epithelial cells, or endothelial cells isolated from the lungs of Stroke animals (Fig. 4).

## Discussion

In the rat model of focal ischemic stroke used herein, V_T_, T_E_, and V_T_/T_I_ increased while RR and T_I_/T_TOT_ decreased during spontaneous breathing, but lung mechanics and gas exchange did not differ during mechanical ventilation. Lungs from stroke rats showed evidence of increased diffuse alveolar damage mainly due to augmented edema and inflammation as shown by increases in total protein levels in BALF. Ultrastructural changes were observed in lung parenchyma, with damage to type 2 pneumocytes and endothelial cells, increased number of macrophages, as well as enlarged basement membrane thickness. Expression of TNF-α and IL-6 in the brain, the TNF-α level in plasma and BALF, as well as the IL-6 level in plasma alone were increased. Additionally, macrophages and endothelial cells, but not epithelial cells, from Stroke animals exhibited increased IL-6 gene expression. In parallel, the phagocytic capability of alveolar macrophages was decreased. Remarkably, all of these changes occurred within 24 h after the induction of cerebral ischemia, which is in agreement with the rapid onset of ALI in stroke patients [7]. In-vitro experiments revealed that the phagocytic capability of alveolar macrophages was reduced and the expression of TNF-α and IL-6 was increased in alveolar macrophages isolated from naïve rats only after exposure to serum from rats that had experienced ischemic stroke.

This is the first experimental study to investigate lung function and histology, systemic inflammation, and the phagocytic capability of alveolar macrophages in an experimental model of focal ischemic stroke. We hypothesized that brain damage would induce systemic inflammation, stimulating alveolar macrophages and triggering a phenotype shift, thus reducing phagocytic capability (Additional file 14: Figure S9). From our data, it seems that specific factors contained in the circulatory compartment following an acute stroke may in turn modulate the innate immune system in the lung. Identification of the factor(s) responsible for these effects (cytokines, alarmins, or other inflammatory mediators) warrants further investigation.

We chose a model of focal, not global, ischemia because focal ischemic stroke has a higher incidence, accounting for approximately 80% of all strokes worldwide [31]. In keeping with previous studies, stroke was induced through thermocoagulation of pial blood vessels over the primary sensorimotor cortices, leading to sensorimotor dysfunction 24 h postoperatively [21]. The fact that peak systolic velocity in the carotid ipsilateral to the lesion decreased after stroke suggests that the regional blood flow in the right cerebral hemisphere was reduced. Following a focal ischemic insult, reperfusion is known to follow a biphasic pattern, with a transient increase (post ischemic hyperperfusion) [32] followed by a more sustained hypoperfusion [33]. As we measured blood flow 24 h after ischemic stroke, our findings likely correspond to the hypoperfusion phase.

Alterations in respiratory pattern following ischemic stroke are common both clinically [34] and experimentally [35], and have been attributed to autonomic dysfunction [36]. In mice, the coefficient of variation for V_T_ and RR increased after an ischemic insult, while the mean values of these variables decreased, leading to reduced minute ventilation [35]. The fact that Stroke animals in our study exhibited increased V_T_ and decreased RR might be due not only to interspecies differences, but also to the severity and location of brain injury as well as central or neurogenic hyperventilation.

Our observation that ischemic strokes are associated with a significant increase in DAD score and lung ultrastructural changes might be explained by higher levels of TNF-α and IL-6 in the plasma and lungs, and of TNF-α in BALF. These data are consistent with experimental [37] and clinical [38] reports of increased systemic inflammation after stroke. The fact that total protein levels in BALF were higher in Stroke animals likely reflects increased permeability of the pulmonary capillary membrane due to inflammation [39, 40]. In turn, elevated inflammatory marker levels in the lungs could have resulted from decompartmentalization of the inflammatory response in the brain, where overexpression of cytokines was detected. Stroke has indeed been shown to disrupt the blood–brain barrier (BBB) [41]. Alternatively, lung inflammation may have been a result of focal ischemia-induced parasympathetic nervous system impairment, leading to loss of the protective cholinergic anti-inflammatory pathway [13, 42, 43]. We cannot rule out that higher V_T_ contributed, at least partly, to increased lung damage and inflammation in Stroke animals. Increased V_T_ has been associated with development of lung injury even in the absence of a first hit [44].

Despite the presence of alveolar edema and bronchoconstriction, these alterations were not sufficient to impair blood gases and lung mechanics. Bronchoconstriction might be explained by increased airway narrowing due to circulating proinflammatory cytokines [45]. In patients with brain injury, bronchoconstriction with increased airway resistance is common [46]. Despite histologic evidence of edema and bronchoconstriction, lung mechanics did not differ between Stroke and Sham animals. One possible explanation is that functional changes are observed only after a certain threshold of pulmonary damage has been exceeded [47]. Additionally, animals were sedated, anesthetized, paralyzed, and mechanically ventilated with PEEP = 3 cmH_2_O, which prevented development of possible lung mechanical changes. Interestingly, in patients with severe brain damage, respiratory mechanics and arterial blood gases differed at ZEEP but not at PEEP = 8 cmH_2_O [46], which is consistent with our results.

Although we are unable to claim increased lung infection susceptibility based on our data, it is possible to speculate that the reduction of alveolar macrophage phagocytic capability that we observed could increase the risk of pneumonia after stroke in the clinical setting. Pneumonia is a common complication of stroke, affecting up to 22% of patients after stroke, and is known to worsen clinical and neurological outcomes [48, 49]. The negative results of two randomized trials of prophylactic antibiotics in stroke [50, 51] indicate that the mechanisms leading to lower respiratory tract infections after stroke need to be elucidated [52]. The pathophysiological processes that result in immunosuppression and gut translocation of bacteria after ischemic stroke have been studied in mice, and are partially dependent on sympathetic nervous system activation [12, 13]. Catecholamine release has also been implicated in neurogenic pulmonary edema after severe brain injury, although it has become evident that other mechanisms might contribute to lung dysfunction, including systemic release of proinflammatory mediators, alarmins, and extracellular vesicles, which may induce lung inflammation and injury [18, 53–55]. The cholinergic anti-inflammatory pathway also seems to be involved in brain–lung crosstalk [13, 42], but the mechanisms remain unclear.

### Possible clinical implications

Our results suggest that focal ischemic stroke may lead to brain–lung crosstalk resulting in increased inflammation in pulmonary tissue. As many stroke patients ultimately require mechanical ventilation, this inflammation might serve as a first hit, and particular attention should be paid to avoid ventilator-induced lung injury.

The reduced alveolar macrophage function associated with focal ischemic stroke can expose patients to a higher risk of pneumonia. In patients who need mechanical ventilation, the risk of ventilator-associated pneumonia might be increased, and prophylactic measures should be considered judiciously.

Importantly, the lack of gas exchange and lung mechanics impairment in focal ischemic stroke should not be interpreted as evidence for an absence of brain–lung crosstalk.

### Limitations

This study has several limitations. First, anesthesia was achieved with ketamine, which is a known bronchodilator and might have masked bronchoconstriction during measurements of lung mechanics. Second, we used a model of focal ischemia, and cannot rule out the possibility that results would differ in other models of brain damage. Third, it must be kept in mind that our model does not reproduce the more complex clinical scenario, and our findings cannot be directly extrapolated to human patients. Fourth, the breathing pattern might be influenced by animal species and size [56], as well as the location and intensity of brain damage [57]; however, we cannot rule out the influence of these factors on lung injury or macrophage function. Fifth, controversial results have been reported concerning the effects of ketamine on intracranial pressure [58, 59], but ketamine is still used, even in the clinical setting, due to its protective effects on hemodynamic responses and lung function [60]. Finally, mechanisms of immunosuppression, which were previously evaluated in experimental models of stroke [5, 6, 13], were not analyzed in our study, but are a future line of investigation in the laboratory.

## Conclusion

In the present study, focal ischemic stroke altered the respiratory pattern, induced histological lung damage and inflammation, and decreased the phagocytic capability of alveolar macrophages, without deterioration of pulmonary function. The mechanism associated with reduced phagocytic capability of alveolar macrophages seems to be related to release of serum rather than BALF mediators. Moreover, IL-6 gene expression was increased both in macrophages and endothelial cells, but not in epithelial cells, isolated from the lungs of Stroke animals. Taken together, these findings suggest dynamic crosstalk between the brain and lungs even after relatively mild/moderate brain injury due to stroke.

## Additional files

Additional file 1: Word file detailing the methods (DOCX 50 kb)

Additional file 2: **Table S1.** Forward and reverse oligonucleotide sequences of target gene primers used in experiments (DOCX 12 kb)

Additional file 3: **Figure S1.** Symmetry score in Sham and focal ischemic stroke (Stroke) rats (DOCX 64 kb)

Additional file 4: **Figure S2.** Representative magnetic resonance images (DOCX 746 kb)

Additional file 5: **Figure S3.** Representative carotid Doppler ultrasound scan from an animal before and after focal ischemic stroke. Carotid peak systolic velocity and resistive index before and after ischemic stroke (DOCX 788 kb)

Additional file 6: **Table S2.** Lung mechanics in Sham and focal ischemic stroke (Stroke) groups (DOCX 12 kb)

Additional file 7: **Table S3.** Arterial blood gas analysis in Sham and focal ischemic stroke (Stroke) groups (DOCX 21 kb)

Additional file 8: **Figure S4.** Representative photomicrographs of lung parenchyma in Sham and Stroke rats (DOCX 326 kb)

Additional file 9: **Figure S5.** Ultrastructural features of the alveolar–capillary barrier in Sham and Stroke rats (DOCX 1660 kb)

Additional file 10: **Table S4.** Semiquantitative analysis of lung electron microscopy in Sham and Stroke rats (DOCX 16 kb)

Additional file 11: **Figure S6.** Real-time polymerase chain reaction analysis of biological markers associated with inflammation (interleukin (IL)-6 and tumor necrosis factor (TNF)-α) in brain (left panels) and lung (right panels) in Sham and Stroke groups. Boxes show interquartile (25–75%) range, whiskers denote range (minimum–maximum), horizontal lines represent median in 6 animals/group (DOCX 2504 kb)

Additional file 12: **Figure S7.** Protein levels of interleukin (IL)-6 and tumor necrosis factor (TNF)-α in bronchoalveolar lavage fluid (BALF) and plasma in Sham and Stroke groups (DOCX 2573 kb)

Additional file 13: **Figure S8.** Total protein in BALF in Sham and Stroke groups (DOCX 1545 kb)

Additional file 14: **Figure S9.** Schematic representation of crosstalk between brain and lung (DOCX 400 kb)

## Abbreviations

BALF: Bronchoalveolar lavage fluid
DAD: Diffuse alveolar damage
DV: Diastolic velocity
ELISA: Enzyme-linked immunosorbent assay
FBS: Fetal bovine serum
FiO_2_: Inspired oxygen fraction
FOV: Field of view
FSE: Fast spin echo
MRI: Magnetic resonance imaging
PD: Proton density
PEEP: Positive end expiratory pressure
PSV: Peak systolic velocity resistive index
$$ PSV-\frac{DV}{PSV} $$: Resistive index
RR: Respiratory rate
RT-PCR: Quantitative real-time reverse transcription polymerase chain reaction
T_E_: Expiratory time
T_I_: Inspiratory time
T_I_/T_TOT_: Duty cycle
T_TOT_: Total time
V_T_: Tidal volume
V_T_/T_I_: Mean inspiratory flow
