# Supplementary material for: Focal ischemic stroke leads to lung injury and reduces alveolar macrophage phagocytic capability in rats
Source: Crit Care. 2018 Oct 5;22:249. doi: 10.1186/s13054-018-2164-0 (PMC6173845; doi:10.1186/s13054-018-2164-0)
Supplement: Supplementary file 1 — Word file detailing the methods (DOCX 50 kb) [file 13054_2018_2164_MOESM1_ESM.docx]

**Additional File 1**

**Detailed Methods**

**Study approval**

This study was approved by the Animal Care Committee of the Health Sciences Center, Federal University of Rio de Janeiro (CEUA: 145/13), and registered with the Brazilian National Council for Animal Experimentation Control. All animals received humane care in compliance with the “Principles of Laboratory Animal Care” formulated by the National Society for Medical Research and the U.S. National Academy of Sciences *Guide for the Care and Use of Laboratory Animals*.

**Animal preparation and experimental protocol**

Forty-eight male Wistar rats (weight 350-400 g) were anesthetized [xylazine 2.5 mg/kg, intraperitoneally (i.p.) and ketamine 75 mg/kg, i.p.] and then randomly allocated to undergo ischemic stroke induction by thermocoagulation of pial vessels over the right primary sensorimotor cortex (Stroke) or sham surgery (Sham). After 24 h, 12 animals underwent the cylinder test for analysis of forelimb use asymmetry and brain magnetic resonance imaging (MRI) to confirm presence of cortical ischemic stroke. Animals were then placed separately in closed chambers for noninvasive plethysmography [[1](#_ENREF_1)] and, at the end of the experiment, lungs were removed for histology. In 12 other rats, invasive lung mechanics was evaluated and BALF, blood, lungs, and brains were harvested for molecular biology analysis. In 12 additional rats, alveolar macrophages from naïve Wistar rats were extracted and then incubated with either serum or BALF collected from Sham or Stroke rats to evaluate phagocytic capability and mRNA expression of interleukin (IL)-6 and tumor necrosis factor (TNF)-α. Finally, 12 additional rats (n=6/group) were used to evaluate protein levels of IL-6 and TNF-α in lung and brain tissue homogenates as well as for isolation of alveolar macrophages, epithelial and endothelial cells from Sham and Stroke animals to analyze the mRNA expression of IL-6 and TNF-α in these cells. At the end of all experiments, animals were euthanized by sodium thiopental overdose (150 mg/kg, i.p.).

**Carotid Doppler ultrasound**

Once anesthesia had been induced, the skin overlying each animal’s neck was shaved and prepared. Rats were then placed on a heating bed (EFF 421, INSIGHT®, Brazil) to maintain body temperature at 37 °C. Carotid Doppler ultrasound was performed using a Samsung UGEO HM70 system (São Paulo, Brazil) with an 8–13 MHz linear transducer. A small amount of echo gel was applied to the neck to improve the conductance of the ultrasound probe. Color flow Doppler was used to locate the arteries and guide probe placement to obtain pulse waveforms. The probe was directed parallel to the blood stream, and the angle was maintained at <20°. The spectral Doppler mode tracing was used to extract the following parameters: peak systolic velocity (PSV), diastolic velocity (DV), and resistive index (PSV – DV/PSV) [[2](#_ENREF_2)].

**Surgery**

Anesthetized rats were placed into a stereotactic frame and their heads were immobilized. Cerebral ischemia was induced by thermocoagulation of the pial blood vessels overlying the primary somatosensory, motor, and sensorimotor cortices, as described elsewhere [[3](#_ENREF_3)]. Briefly, the skull was surgically exposed and a craniotomy was performed, exposing the left frontoparietal cortex (+2 to −6 mm A.P. from the bregma). The blood in the pial vessels was thermocoagulated transdurally by bringing a hot probe close to the dura mater. The color of the blood vessels is normally light red; we considered thermocoagulation complete once they had turned dark red. Care was taken to avoid touching or tearing the dura mater. After the procedure, the skin was sutured (Nylon 3-0, ETHICON®, Germany), and the animals were kept warm using a heating bed and returned to the colony room to recover from anesthesia. Sham surgeries were performed using the same procedure, but without thermocoagulation. After the procedures, all animals had access to water and food ad libitum.

**Cylinder test**

At 24 hours, animals underwent the cylinder test for assessment of forelimb use asymmetry [[4](#_ENREF_4)]. The test consists of placing the animal inside a glass cylinder (diameter 20 cm, height 30 cm) and counting each instance of use of the ipsilateral (to the lesion) forelimb alone, contralateral forelimb alone, or simultaneous use of both forelimbs. For each animal, the percentage relative to the total number of uses (ipsilateral + contralateral + simultaneous) was calculated for the ipsilateral (unimpaired) uses and contralateral (impaired) uses. Then, an asymmetry score was calculated for each animal by the following formula: asymmetry score = (% of ipsilateral uses) − (% of contralateral uses). The asymmetry score was then converted to a symmetry score (100 − asymmetry score).

**Magnetic resonance imaging**

Twenty-four hours after induction of ischemic stroke, one rat underwent MRI at the National Center of Structural Biology and Bioimaging (CENABIO/UFRJ). For MRI, the rat was anesthetized and positioned in the MRI coil. Images were acquired in a 7-T magnetic resonance scanner (7T/210 horizontal Varian scanner, Agilent Technologies, Palo Alto, CA, USA), using fast spin echo (FSE) proton density (PD) sequences (matrix: 192×192, slice thickness: 0.5 mm; 15 continuous slices) in the axial (TR/TE: 1500/11 ms; field of view: 30×30 cm), coronal (TR/TE: 2100/11 ms; field of view: 30×30.5 cm), and sagittal (TR/TE: 1500/11 ms; field of view: 30×30.5 cm) planes. Data were processed in VnmrJ software (Agilent Technologies), and an observational analysis was performed.

**Respiratory parameters**

Conscious, spontaneously breathing rats were placed in a whole-body plethysmography system (FinePointe™ R/C Buxco; Buxco Eletronics, Sharon, CT, USA). Flow through each chamber was maintained at 1.5 L/min; Buxco air flow transducers (TRD5700) were connected to the chambers and to a reference chamber to compensate for pressure changes. The following parameters were measured: tidal volume (V_T_), respiratory rate (RR), inspiratory (T_I_), expiratory (T_E_), and total (T_TOT_) time, mean inspiratory flow (V_T_/T_I_), and duty cycle (T_I_/T_TOT_) [[5](#_ENREF_5)].

**Invasive lung mechanics**

After sedation, anesthesia, tracheostomy, and neuromuscular blockade with vecuronium bromide (2 mg/kg i.v.), animals were mechanically ventilated (Servo-I, MAQUET, Solna, Sweden) in volume-controlled mode, with V_T_ = 6 mL/kg, RR = 80 breaths/min, fraction of inspired oxygen (FiO_2_) = 0.4, and 3 cmH_2_O of positive end-expiratory pressure (PEEP) for 5 minutes. Airflow, airway pressure (Paw), and esophageal pressure (Pes) were recorded continuously throughout the experiments on a computer running custom-made software written in LabVIEW (National Instruments, Austin, TX). V_T_ was calculated by digital integration of the airflow signal. Transpulmonary pressure (P,L) was calculated during inspiration and expiration as the difference between Paw and Pes. Airway resistance and lung static elastance were computed by the end-inflation occlusion method [[6](#_ENREF_6)]. Briefly, after end-inspiratory occlusion, there is an initial rapid drop in pressure from the pre-occlusion value (peak inspiratory pressure) down to an inflection point (∆P1), followed by slow pressure decay until a plateau (Pplat,L) is reached. This plateau corresponds to the elastic recoil pressure of the lung (L). Airway resistance (Raw) was calculated by dividing ∆P1 by airflow, whereas Est,L was calculated by dividing Pplat,L by tidal volume. Lung mechanics measurements were performed 10 times in each animal. All signals were amplified in a four-channel signal conditioner (SC-24, SCIREQ, Montreal, QC, Canada), and sampled at 200 Hz with a 12-bit analog-to-digital converter (National Instruments, Austin, Texas, USA). All mechanical data were computed offline by a routine written in MATLAB (Version R2007a, The Mathworks Inc., Natick, Massachusetts, USA).

**Bronchoalveolar lavage fluid (BALF)**

After removal of lungs at PEEP = 3 cmH_2_O, a polyethylene cannula was inserted into the left bronchus and phosphate-buffered saline solution was instilled (1.5 ml, at 37 °C) and aspirated three times. Samples were centrifuged at 1500*g* for 10 minutes, at a temperature of 4 °C, then stored at −80 °C for further analysis. Protein content was determined by the Bradford method [[7](#_ENREF_7)].

**Phagocytic capability of alveolar macrophages**

The phagocytic capability of alveolar macrophages was tested with pH-sensitive pHrodo™ Green Zymosan A BioParticles^®^ (Life Technologies, Carlsbad, CA, USA) conjugate for phagocytosis, as per supplier instructions. pHrodo® Green conjugates are non-fluorescent outside the cell at neutral pH, but fluoresce brightly green at acidic pH, such as in phagosomes. Cells collected from BALF were seeded on a tissue culture dish and incubated in RPMI1640 10% fetal bovine serum (FBS) with 1% penicillin/streptomycin for 2 h at 37 °C, 5% CO_2._ Cells that adhered to the plate are macrophages [[8](#_ENREF_8)]*.* Briefly, a total of 10^5^ alveolar macrophages were plated on a 96-well plate. Cells were washed with saline (0.9% NaCl) and incubated with fluorescently pHrodo-Green-labeled *S. cerevisiae* particles (0.5 mg/mL) for 2 h. After incubation, the cells were placed on ice to halt phagocytosis, washed twice with ice-cold PBS, and prepared for analysis. Fluorescence was measured in a microplate reader (Perkin-Elmer, Waltham, USA). Phagocytosis of fluorescently labeled BioParticles was quantified by measuring intracellular fluorescence emitted by engulfed particles at 585 nm.

**Lung histology**

The right lung was fixed in 4% buffered formalin and embedded in paraffin. Sections (3 μm thick) were cut longitudinally from the central zone with a microtome and stained with hematoxylin-eosin for histological analysis. Photomicrographs at magnifications of ×25, ×100, and ×400 were obtained from eight non-overlapping fields of view per section using a light microscope (Olympus BX51, Olympus Latin America Inc., Brazil). Diffuse alveolar damage (DAD) and the degree of bronchoconstriction were quantified by an expert in lung pathology (VLC) blinded to group assignment, using a weighted scoring system, as previously described [[9](#_ENREF_9)]. To evaluate DAD, the following histological features were analyzed in lung tissue: interstitial edema, inflammatory infiltration, and atelectasis. Values from 0 to 4 were used to represent the severity of a given feature, with 0 standing for no effect and 4 for maximum severity. In addition, the extent of involvement in each field of view was also determined on a scale of 0 to 4, with 0 standing for no appearance and 4 for complete involvement. Scores were calculated as the product of severity and extent of each feature, ranging from 0 to 16, whereas the total DAD score was the sum of these three features (from 0 to 48).

**Transmission electron microscopy**

Three slices (2 × 2 × 2 mm) were cut from three different segments of the left lung for electron microscopy. On each lung electron microscopy image (20 fields of view per animal), damage to the alveolar–capillary membrane, type 2 epithelial cells and endothelial cells, basement membrane thickness, macrophages, and degree of interstitial edema were graded on a five-point, semiquantitative, severity-based scoring system as follows: 0 = normal lung parenchyma, 1 = changes in 1 to 25% of examined tissue, 2 = 26 to 50% of examined tissue, 3 = 51 to 75% of examined tissue, and 4 = 76 to 100% of examined tissue [[6](#_ENREF_6)].

**Alveolar macrophage and endothelial and epithelial cell isolation**

Rats were euthanized with a lethal dose of thiopental and the heart and lungs were removed *en bloc*. The right lung was used for epithelial-cell isolation, and the left lung, for endothelial-cell and macrophage isolation. The left lung was isolated from the heart-lungs block, the trachea was attached to a cannula, and the right lung was washed through the trachea and through right ventricle with PBS, followed by PBS with EGTA at room temperature (RT). The heart was removed and the lungs filled with elastase solution (4 IU/mL elastase in Ham’s F12) for 30 min, then cut into pieces and transferred into Digestion solution (DNAse 0.5 mg/mL, bovine serum albumin 0.01 mg/mL, 1% penicillin–streptomycin in Ham’s F12 medium) for 15 minutes at 37 °C. Epithelial cells were dissociated by gentle agitation, pooled, washed, and incubated in HBSS solution (amphotericin B 12.5 μg/mL, EDTA 0.1 M, 10 mM HEPES, 2% FBS, 1% penicillin–streptomycin in Hank’s Buffered Salt Solution) to remove non-epithelial cells. The remaining solution was transferred to a 70-μm cell strainer, the reaction was stopped with adult bovine serum, and the solution spun at 600*g* at 4 °C. Cells were resuspended in red blood cell lysis buffer for 1 min, washed with HBSS solution, and spun at 600*g* at 4 °C. Epithelial cells were isolated from the resulting pellet and used for RNA extraction.

Endothelial cells and macrophages were isolated by magnetic sorting. The left lung was cut into small pieces and digested with collagenase 1% for 40 min under mild agitation. Proteolysis was stopped with DMEM with 10% FBS, centrifuged at 600*g*/4 °C. The pellet was resuspended in PBS containing biotinylated anti-CD54 (endothelial-cell) and anti-CD11b+ (monocyte) antibodies on ice for 30 min. Cells were washed with PBS and the incubated with Dynabeads biotin binder (Thermofisher) as per manufacturer instructions, for 20 min, on ice. Cells were then isolated through exposure to a magnetic field.

**Real-time PCR**

Quantitative real-time reverse transcription polymerase chain reaction (RT-PCR) was performed to measure biological markers associated with inflammation—interleukin (IL)-6, tumor necrosis factor (TNF)-α—and a housekeeping gene (acidic ribosomal phosphoprotein P0, *36B4*) in the brain and left lung. Central slices of the left lung and peri-lesion slices of the brain were cut, collected in cryotubes, flash-frozen by immersion in liquid nitrogen, and stored at −80 °C. Total RNA was extracted from frozen tissues or from isolated cells (epithelial, endothelial and macrophages) using a RNeasy plus® mini kit (Qiagen, USA), following the manufacturer’s recommendations. RNA concentration was measured by spectrophotometry in a Nanodrop ND-1000 system (Thermo Fisher Scientific, USA). First-strand complementary DNA was synthesized from total RNA using the QuantiTect® Reverse Transcription Kit (Qiagen, USA). The primers used are listed in Additional File 2. Relative mRNA levels were measured with a BRYT Green system (Promega, Fitchburg, WI) using a Mastercycler ep Realplex thermal cycler (Eppendorf, Hamburg, Germany). For each sample measured in triplicate, gene expression was normalized to that of *36B4* [[10](#_ENREF_10)] and expressed as fold change relative to Sham animals, using the 2^−ΔΔ^Ct method, where ΔCt = Ct (target gene) minus Ct (reference gene). This is a suitable method to analyze relative changes in gene expression from quantitative RT-PCR experiments.

**Enzyme-linked immunosorbent assay (ELISA)**

IL-6 and TNF-α levels were quantified in BALF, lung, brain, and plasma by ELISA. Procedures were done as per the manufacturer’s protocol (PeproTech, London, UK) and normalized to total protein as assessed by Bradford’s method (Sigma-Aldrich, St Louis, MO, USA) [[11](#_ENREF_11)].

**Statistical analysis**

Sample size calculation was based on effect estimates obtained from previous studies in rodents using similar settings [[12](#_ENREF_12)]. A sample size of eight animals per group (providing for one animal as dropout) would provide the appropriate power (1-β = 0.8) to identify significant (α = 0.05) differences in the symmetry score, considering an effect size d = 1.9, a two-sided test, and a sample size ratio = 1 (G*Power 3.1.9.2, University of Düsseldorf, Germany).

The Kolmogorov-Smirnov test with Lilliefors’ correction was used to assess normality of data, while the Levene median test was used to evaluate the homogeneity of variances. The *t*-test and Mann-Whitney *U* test were used for parametric and nonparametric data respectively. Parametric data are expressed as mean ± SD, and nonparametric data as median (interquartile range). All tests were carried out in GraphPad Prism v6.07 (GraphPad Software, La Jolla, California, USA). Significance was established at p<0.05. All assessments were performed by investigators blinded to group assignment.

**References**

1. Rocco PR, Faffe DS, Feijóo M, Menezes SL, Vasconcellos FP, et al. Effects of uni- and bilateral phrenicotomy on active and passive respiratory mechanics in rats. Respir Physiol 1997; 110: 9-18.
2. Morgan EE, Casabianca AB, Khouri SJ, Kalinoski AL. In vivo assessment of arterial stiffness in the isoflurane anesthetized spontaneously hypertensive rat. Cardiovasc Ultrasound. 2014;12:37 doi: 10.1186/1476-7120-12-37.
3. Giraldi-Guimardes A, Rezende-Lima M, Bruno FP, Mendez-Otero R. Treatment with bone marrow mononuclear cells induces functional recovery and decreases neurodegeneration after sensorimotor cortical ischemia in rats. Brain Res. 2009;1266:108-20.
4. Schallert T. Behavioral tests for preclinical intervention assessment. NeuroRx : 2006,3:497-504.
5. Hoymann HG. Invasive and noninvasive lung function measurements in rodents. J Pharmacol Toxicol Methods. 2007;55:16-26.
6. Riva DR, Oliveira MB, Rzezinski AF, Rangel G, Capelozzi VL, et al. Recruitment maneuver in pulmonary and extrapulmonary experimental acute lung injury. Crit Care Med. 2008;36:1900-8.
7. Olson BJ, Markwell J. Assays for determination of protein concentration. Curr Protoc Protein Sci 2007; Chapter 3:Unit 3.4  doi: 10.1002/0471140864.ps0304s48.
8. Herre J, Marshall AS, Caron E, Edwards AD, Williams DL, et al. [Dectin-1 uses novel mechanisms for yeast phagocytosis in macrophages.](https://www.ncbi.nlm.nih.gov/pubmed/15304394) Blood. 2004;104: 4038-45
9. Uhlig C, Silva PL, Ornellas D, Santos RS, Miranda PJ, et al. The effects of salbutamol on epithelial ion channels depend on the etiology of acute respiratory distress syndrome but not the route of administration. Respir Res. 2014;15:56 doi: 10.1186/1465-9921-15-56.
10. Schmittgen TD, Livak KJ. Analyzing real-time PCR data by the comparative C(T) method. Nat Protoc. 2008;3:1101-8.
11. Chao MC, Garcia CS, de Oliveira MB, Santos RS, Lucas IH, et al. Degree of endothelium injury promotes fibroelastogenesis in experimental acute lung injury. Respir Physiol Neurobiol. 2010;173:179-88.
12. de Vasconcelos Dos Santos A, da Costa Reis J, Diaz Paredes B, Moraes L, Jasmin, et al. Therapeutic window for treatment of cortical ischemia with bone marrow-derived cells in rats. Brain Res. 2010;1306:149-58.
