# Supplementary material for: Focal ischemic stroke leads to lung injury and reduces alveolar macrophage phagocytic capability in rats
Source: Crit Care. 2018 Oct 5;22:249. doi: 10.1186/s13054-018-2164-0 (PMC6173845; doi:10.1186/s13054-018-2164-0)
Supplement: Supplementary file 2 — Table S1. Forward and reverse oligonucleotide sequences of target gene primers used in experiments (DOCX 12 kb) [file 13054_2018_2164_MOESM2_ESM.docx]

**Additional File 2**

**Table S1**. Forward and reverse oligonucleotide sequences of target gene primers used in experiments.

| **Gene** | **Primer** | **Primer sequences (5′-3′)** |
| --- | --- | --- |
| IL-6 | Forward | CTCCGCAAGAGACTTCCAG |
|  | Reverse | CTCCTCTCCGGACTTGTGA |
| TNF-α | Forward | TGCCTTCTTCCCTGTTCC |
|  | Reverse | CTGGGCAGCGTTTATTCT |
| *36B4* | Forward | AATCCTGAGCGATGTGCAG |
|  | Reverse | GCTGCCATTGTCAAACAC |

IL-6, interleukin-6; TNF-α, tumor necrosis factor alpha; *36B4*, acidic ribosomal phosphoprotein P0.
