# Supplementary material for: Focal ischemic stroke leads to lung injury and reduces alveolar macrophage phagocytic capability in rats
Source: Crit Care. 2018 Oct 5;22:249. doi: 10.1186/s13054-018-2164-0 (PMC6173845; doi:10.1186/s13054-018-2164-0)
Supplement: Supplementary file 3 — Figure S1. Symmetry score in Sham and focal ischemic stroke (Stroke) rats (DOCX 64 kb) [file 13054_2018_2164_MOESM3_ESM.docx]

**Additional File 3**

**
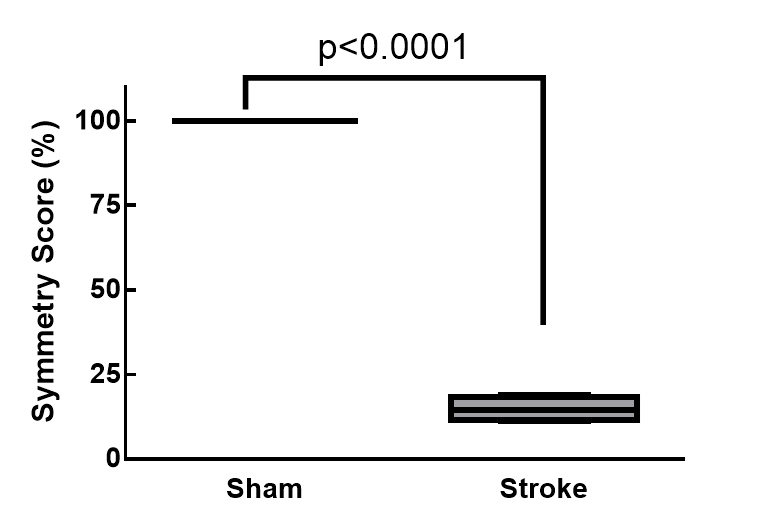
**

**Figure S1**. Symmetry score in Sham and focal ischemic stroke (Stroke) rats. Boxes show the interquartile (25–75%) range, whiskers encompass the range (minimum–maximum), and horizontal lines represent the median in 6 animals/group.
