# Supplementary material for: Focal ischemic stroke leads to lung injury and reduces alveolar macrophage phagocytic capability in rats
Source: Crit Care. 2018 Oct 5;22:249. doi: 10.1186/s13054-018-2164-0 (PMC6173845; doi:10.1186/s13054-018-2164-0)
Supplement: Supplementary file 4 — Figure S2. Representative magnetic resonance images (DOCX 746 kb) [file 13054_2018_2164_MOESM4_ESM.docx]

**Additional File 4**


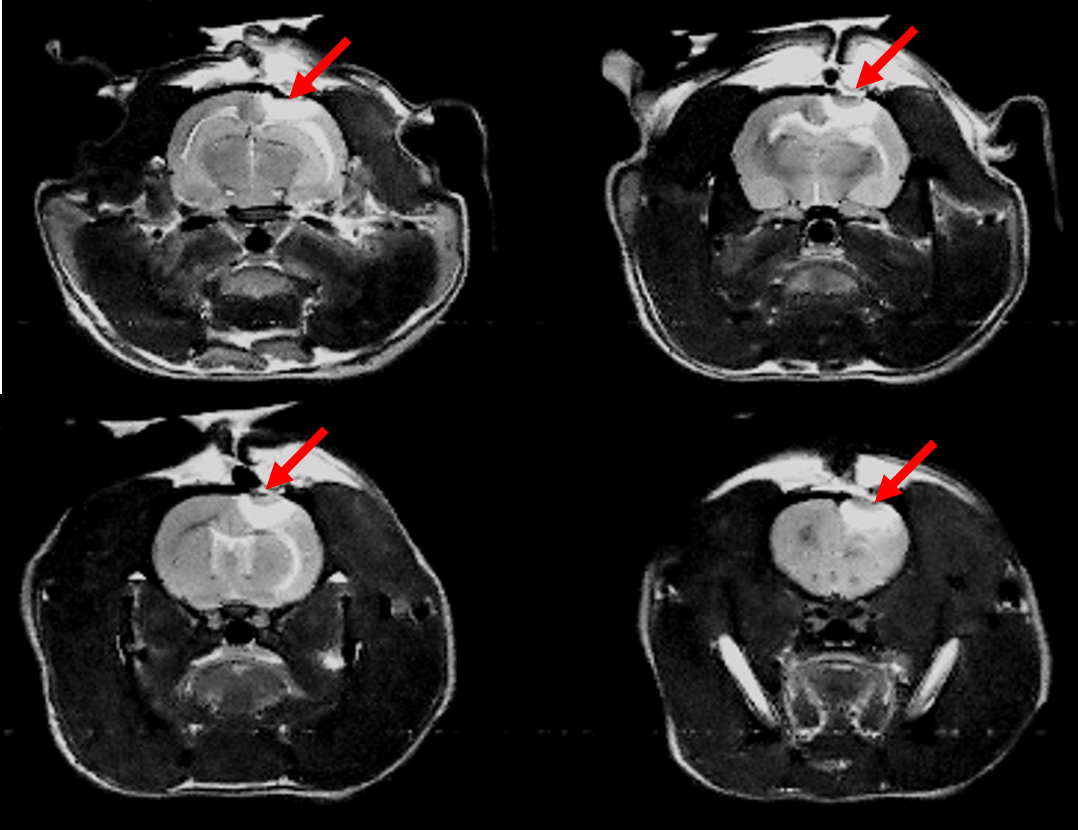


**Figure S2**. Representative magnetic resonance images. Axial FLAIR scans show an infarct involving the left frontoparietal cortex (red arrows) in an animal 24 hours after ischemic stroke. The area of involvement corresponds to the primary somatosensory, motor, and sensorimotor cortices.
