# Supplementary material for: Focal ischemic stroke leads to lung injury and reduces alveolar macrophage phagocytic capability in rats
Source: Crit Care. 2018 Oct 5;22:249. doi: 10.1186/s13054-018-2164-0 (PMC6173845; doi:10.1186/s13054-018-2164-0)
Supplement: Supplementary file 5 — Figure S3. Representative carotid Doppler ultrasound scan from an animal before and after focal ischemic stroke. Carotid peak systolic velocity and resistive index before and after ischemic stroke (DOCX 788 kb) [file 13054_2018_2164_MOESM5_ESM.docx]

**Additional File 5**


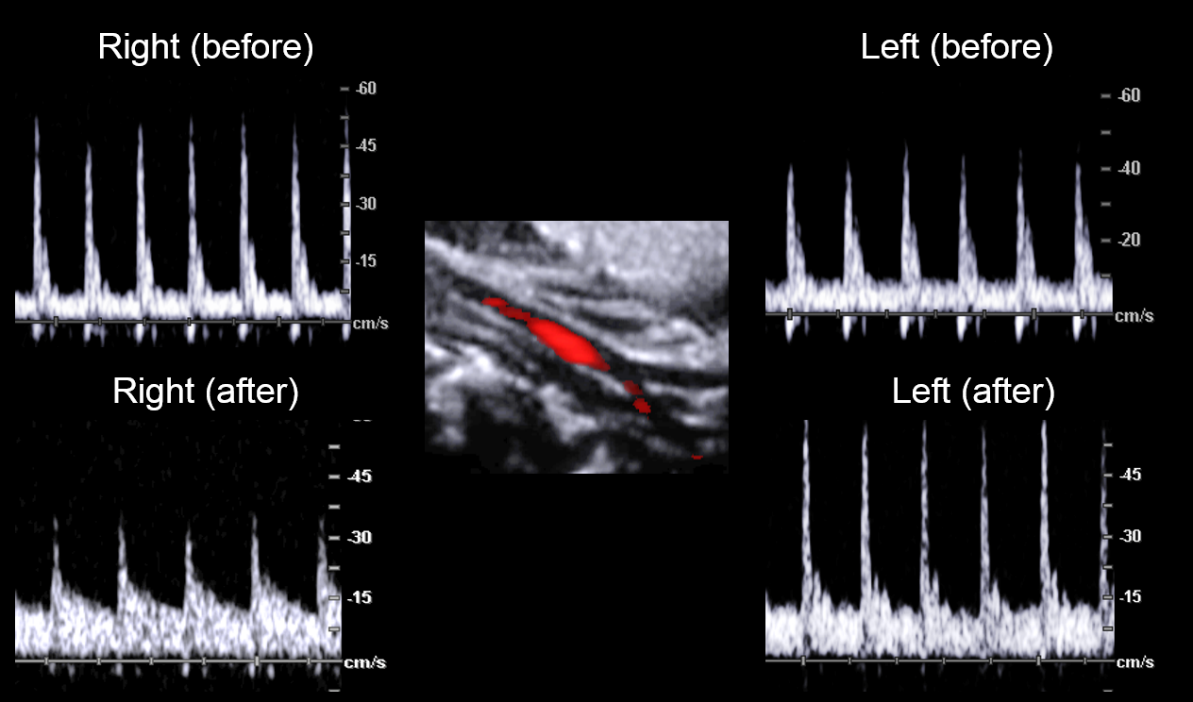


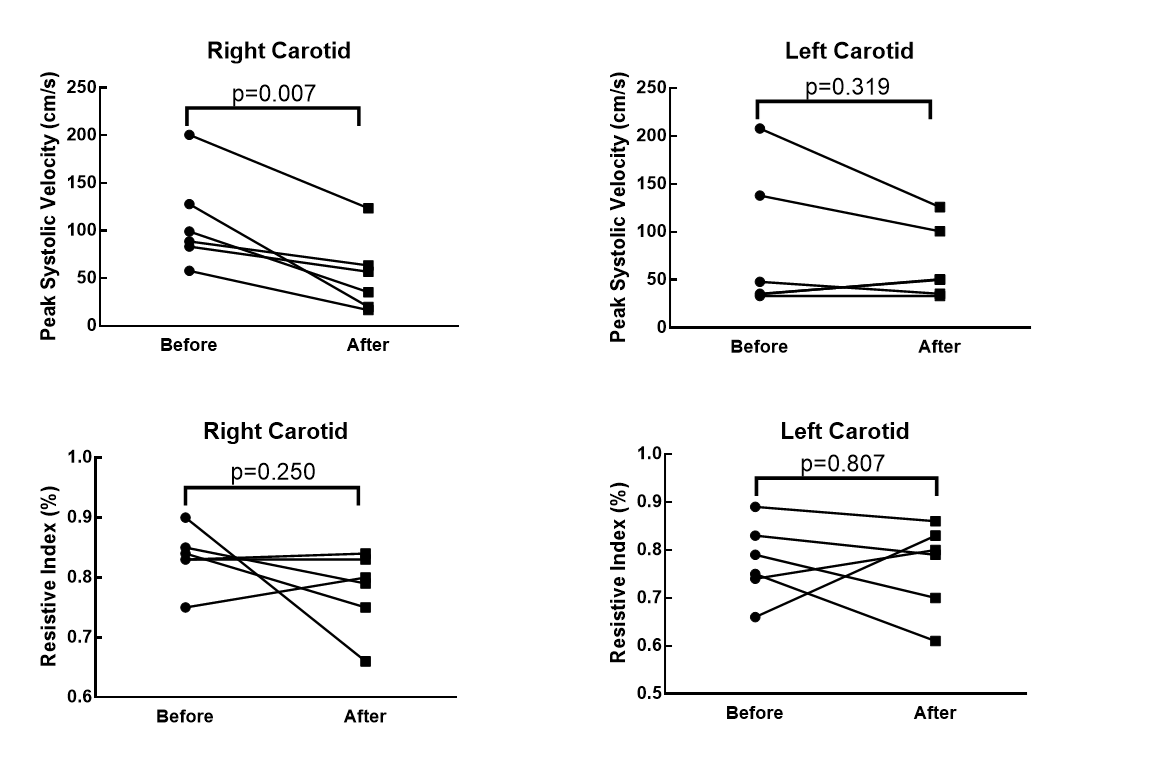


**Figure S3.** Representative carotid Doppler ultrasound scan from an animal before and after focal ischemic stroke induction. Carotid peak systolic velocity and resistive index before and after ischemic stroke. Each symbol represents one animal.
