# Supplementary material for: Focal ischemic stroke leads to lung injury and reduces alveolar macrophage phagocytic capability in rats
Source: Crit Care. 2018 Oct 5;22:249. doi: 10.1186/s13054-018-2164-0 (PMC6173845; doi:10.1186/s13054-018-2164-0)
Supplement: Supplementary file 6 — Table S2. Lung mechanics in Sham and focal ischemic stroke (Stroke) groups (DOCX 12 kb) [file 13054_2018_2164_MOESM6_ESM.docx]

**Additional File 6**

**Table S2.** Lung mechanics in Sham and focal ischemic stroke (Stroke) rats.

|  | **Est,L (cmH_2_O.mL^-1^)** | **Raw (cmH_2_O.mL^-1^.s)** |
| --- | --- | --- |
| **Sham** | 2.8 ± 0.6 | 0.2 ± 0.3 |
| **Stroke** | 3.1 ± 0.6 | 0.2 ± 0.4 |

Values expressed as means ± SD of 6 animals/group. Est,L: static lung elastance; Raw: airway resistance.
