# Supplementary material for: Focal ischemic stroke leads to lung injury and reduces alveolar macrophage phagocytic capability in rats
Source: Crit Care. 2018 Oct 5;22:249. doi: 10.1186/s13054-018-2164-0 (PMC6173845; doi:10.1186/s13054-018-2164-0)
Supplement: Supplementary file 7 — Table S3. Arterial blood gas analysis in Sham and focal ischemic stroke (Stroke) groups (DOCX 21 kb) [file 13054_2018_2164_MOESM7_ESM.docx]

**Additional File 7**

**Table S3.** Arterial blood gas analysis in Sham and focal ischemic stroke (Stroke) rats.

|  | **pHa** | **PaCO_2_ (mmHg)** | **PaO_2_/FiO_2_ (mmHg)** | **HCO_3_^−^ (mmol/L)** |
| --- | --- | --- | --- | --- |
| **Sham** | 7.44 ± 0.03 | 31 ± 6 | 353 ± 47 | 21.9 ± 3.0 |
| **Stroke** | 7.43 ± 0.07 | 33 ± 7 | 336 ± 42 | 22.3 ± 3.9 |

Values expressed as means ± SD of 6 animals/group. pHa: arterial pH; PaCO_2_: partial pressure of arterial carbon dioxide; PaO_2_/FiO_2_: partial pressure of arterial oxygen divided by fraction of inspired oxygen.
