# Supplementary material for: Focal ischemic stroke leads to lung injury and reduces alveolar macrophage phagocytic capability in rats
Source: Crit Care. 2018 Oct 5;22:249. doi: 10.1186/s13054-018-2164-0 (PMC6173845; doi:10.1186/s13054-018-2164-0)
Supplement: Supplementary file 8 — Figure S4. Representative photomicrographs of lung parenchyma in Sham and Stroke rats (DOCX 326 kb) [file 13054_2018_2164_MOESM8_ESM.docx]

**Additional File 8**


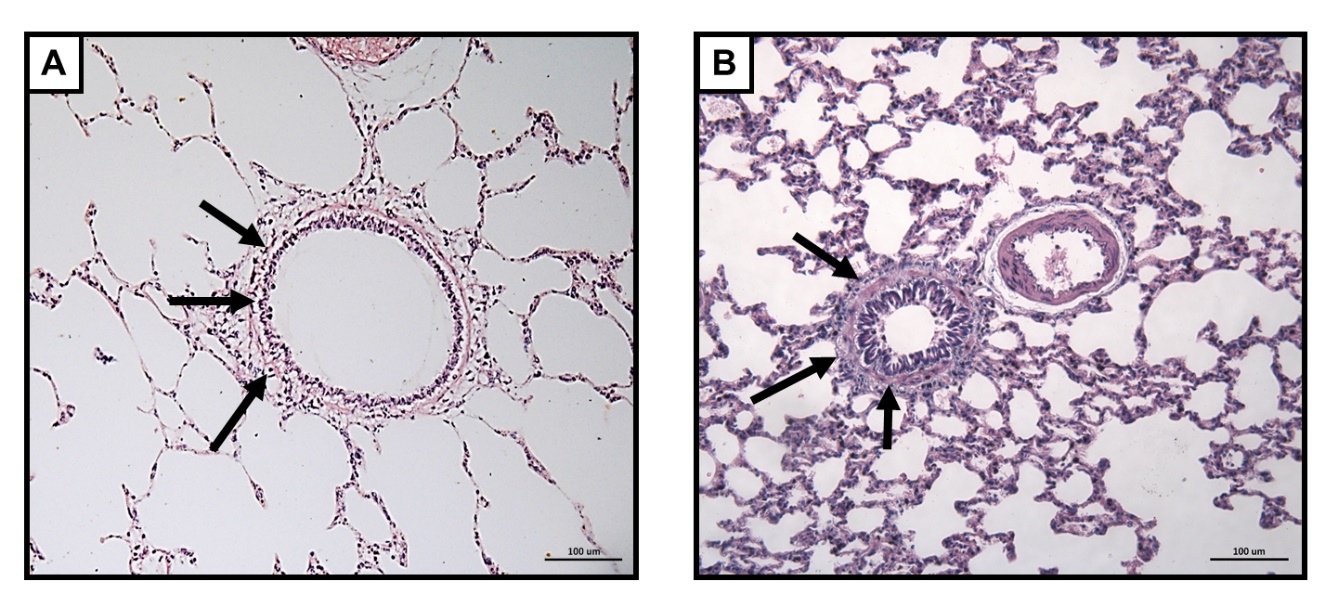


**Figure S4.** Representative photomicrographs of lung parenchyma in Sham (Panel A) and Stroke (Panel B) rats. Hematoxylin-eosin stain, original magnification ×200. Note area of bronchoconstriction in Panel B (arrows).
