# Supplementary material for: Focal ischemic stroke leads to lung injury and reduces alveolar macrophage phagocytic capability in rats
Source: Crit Care. 2018 Oct 5;22:249. doi: 10.1186/s13054-018-2164-0 (PMC6173845; doi:10.1186/s13054-018-2164-0)
Supplement: Supplementary file 9 — Figure S5. Ultrastructural features of the alveolar–capillary barrier in Sham and Stroke rats (DOCX 1660 kb) [file 13054_2018_2164_MOESM9_ESM.docx]

**Additional File 9**


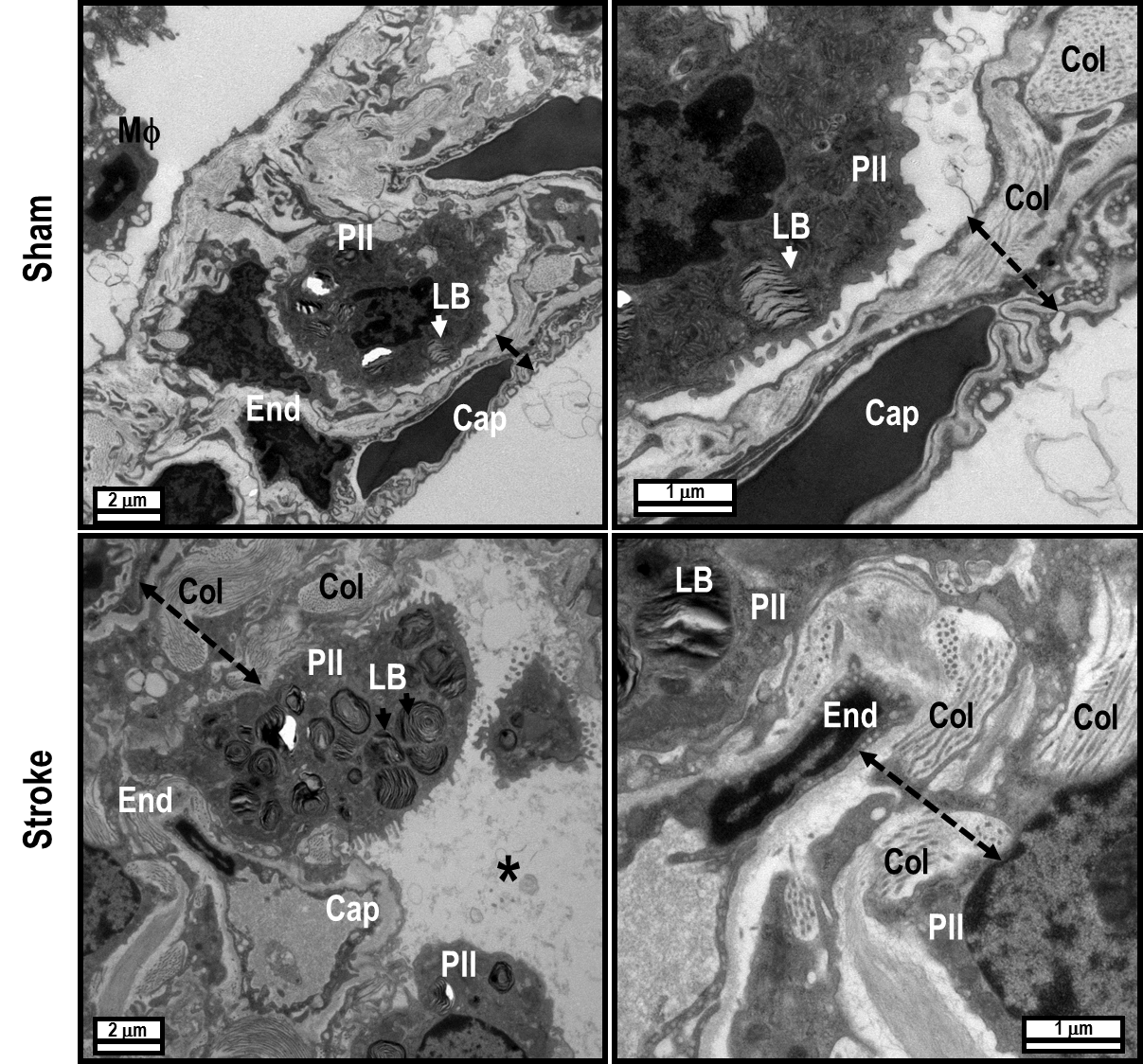


**Figure S5.** Ultrastructural features of the alveolar–capillary barrier in Sham and Stroke animals examined under different magnifications. The Sham slide shows a preserved alveolar–capillary barrier with harmonic distribution of type 2 pneumocytes (PII), endothelial cells (End), and basement membrane thickness (double arrows); note sparse fibers of type III collagen (Col). In contrast, the Stroke animal exhibits prominent intra-alveolar edema (*), increased number of macrophages (Mφ), apoptosis of endothelial cells, disarrangement of lamellar bodies (LB) in PII, and increased basement membrane thickness (double arrows); note prominent type III collagen fibers. Cap: capillary.
