# Supplementary material for: Focal ischemic stroke leads to lung injury and reduces alveolar macrophage phagocytic capability in rats
Source: Crit Care. 2018 Oct 5;22:249. doi: 10.1186/s13054-018-2164-0 (PMC6173845; doi:10.1186/s13054-018-2164-0)
Supplement: Supplementary file 10 — Table S4. Semiquantitative analysis of lung electron microscopy in Sham and Stroke rats (DOCX 16 kb) [file 13054_2018_2164_MOESM10_ESM.docx]

**Additional File 10**

**Table S4.** Semiquantitative analysis of lung electron microscopy in Sham and Stroke rats.

| **Parameters** | **Sham** | **Stroke** |
| --- | --- | --- |
| Edema | 0.5 (0.0-1.0) | 2.0 (1.25-2.75)^*^ |
| Type II epithelial cell damage | 1.0 (0.25-1.0) | 2.0 (1.25-2.0)^*^ |
| Endothelial cell damage | 0.5 (0.0-1.0) | 2.5 (2.0-3.0)^*^ |
| Basement membrane thickness | 1.0 (0.25-1.0) | 2.0 (2.0-2.75)^*^ |
| Increased number of macrophages | 1.0 (1.0-1.0) | 3.0 (2.25-3.0)^*^ |
| Increased collagen fiber content | 1.0 (1.0-1.0) | 2.0 (2.0-2.0) |

Pathologic findings were graded on a five-point, semiquantitative severity-based scoring system: 0=normal lung parenchyma, 1=changes in 1 to 25%, 2=26 to 50%, 3=51 to 75%, and 4=76 to 100% of examined tissue. Values are median (interquartile range) of 6 animals per group. ^*^p<0.05 *vs.* Sham.
