# Supplementary material for: Focal ischemic stroke leads to lung injury and reduces alveolar macrophage phagocytic capability in rats
Source: Crit Care. 2018 Oct 5;22:249. doi: 10.1186/s13054-018-2164-0 (PMC6173845; doi:10.1186/s13054-018-2164-0)
Supplement: Supplementary file 11 — Figure S6. Real-time polymerase chain reaction analysis of biological markers associated with inflammation (interleukin (IL)-6 and tumor necrosis factor (TNF)-α) in brain (left panels) and lung (right panels) in Sham and Stroke groups. Boxes show interquartile (25–75%) range, whiskers denote range (minimum–maximum), horizontal lines represent median in 6 animals/group (DOCX 2504 kb) [file 13054_2018_2164_MOESM11_ESM.docx]

**Additional File 11**


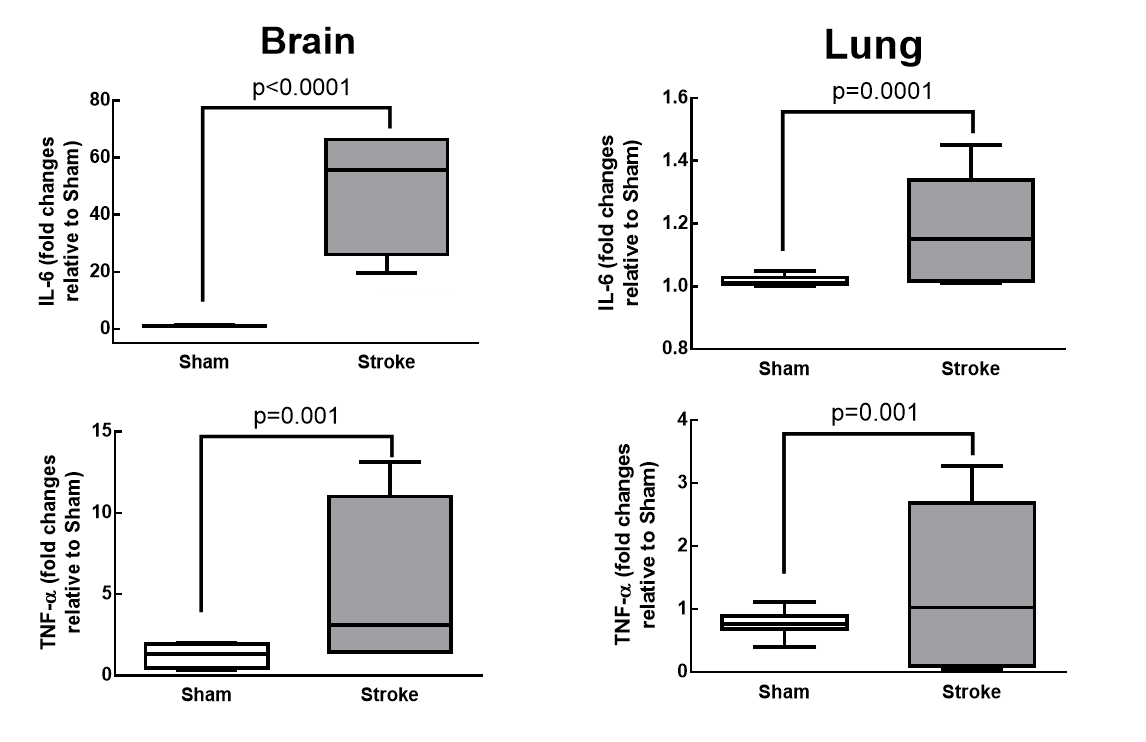


**Figure S6.** Real-time polymerase chain reaction analysis of biological markers associated with inflammation [interleukin (IL)-6 and tumor necrosis factor (TNF)-α] in the brain (left panels) and lung (right panels) of animals in the Sham and Stroke groups. Boxes show the interquartile (25–75%) range, whiskers denote the range (minimum–maximum), and horizontal lines represent the median in 6 animals/group.
