# Supplementary material for: Focal ischemic stroke leads to lung injury and reduces alveolar macrophage phagocytic capability in rats
Source: Crit Care. 2018 Oct 5;22:249. doi: 10.1186/s13054-018-2164-0 (PMC6173845; doi:10.1186/s13054-018-2164-0)
Supplement: Supplementary file 12 — Figure S7. Protein levels of interleukin (IL)-6 and tumor necrosis factor (TNF)-α in bronchoalveolar lavage fluid (BALF) and plasma in Sham and Stroke groups (DOCX 2573 kb) [file 13054_2018_2164_MOESM12_ESM.docx]

**Additional File 12**


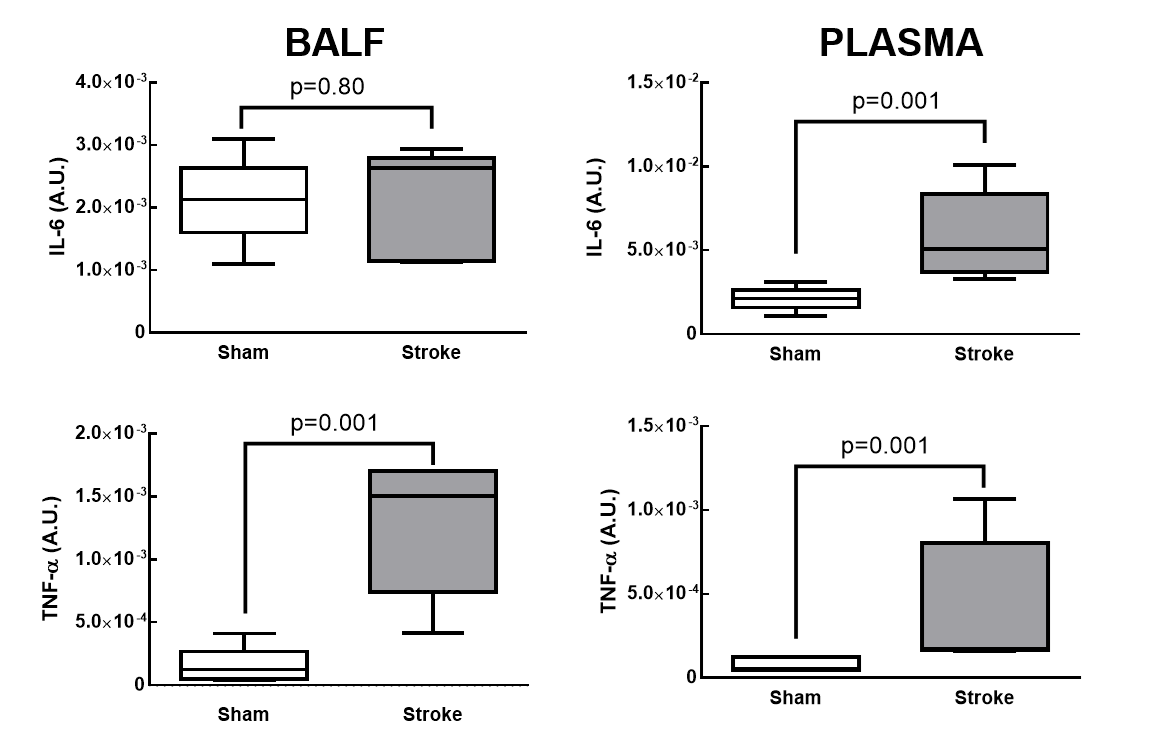


**Figure S6.** Protein levels of interleukin (IL)-6 and tumor necrosis factor (TNF)-α in bronchoalveolar lavage fluid (BALF) and plasma in the Sham and Stroke groups. Boxes show the interquartile (25–75%) range, whiskers denote the range (minimum–maximum), and horizontal lines represent the median in 6 animals/group.
