# Supplementary material for: Focal ischemic stroke leads to lung injury and reduces alveolar macrophage phagocytic capability in rats
Source: Crit Care. 2018 Oct 5;22:249. doi: 10.1186/s13054-018-2164-0 (PMC6173845; doi:10.1186/s13054-018-2164-0)
Supplement: Supplementary file 13 — Figure S8. Total protein in BALF in Sham and Stroke groups (DOCX 1545 kb) [file 13054_2018_2164_MOESM13_ESM.docx]

**Additional File 13**


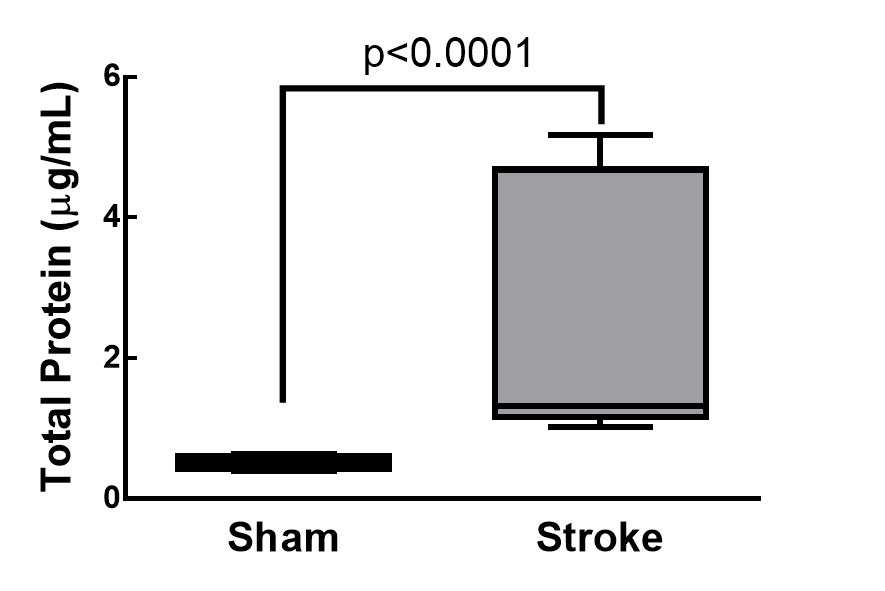


**Figure S8**. Total protein in bronchoalveolar lavage fluid (BALF) in the Sham and Stroke groups. Boxes show the interquartile (25–75%) range, whiskers denote the range (minimum–maximum), and horizontal lines represent the median in 6 animals/group.
