# Supplementary material for: Focal ischemic stroke leads to lung injury and reduces alveolar macrophage phagocytic capability in rats
Source: Crit Care. 2018 Oct 5;22:249. doi: 10.1186/s13054-018-2164-0 (PMC6173845; doi:10.1186/s13054-018-2164-0)
Supplement: Supplementary file 14 — Figure S9. Schematic representation of crosstalk between brain and lung (DOCX 400 kb) [file 13054_2018_2164_MOESM14_ESM.docx]

**Additional File 14**


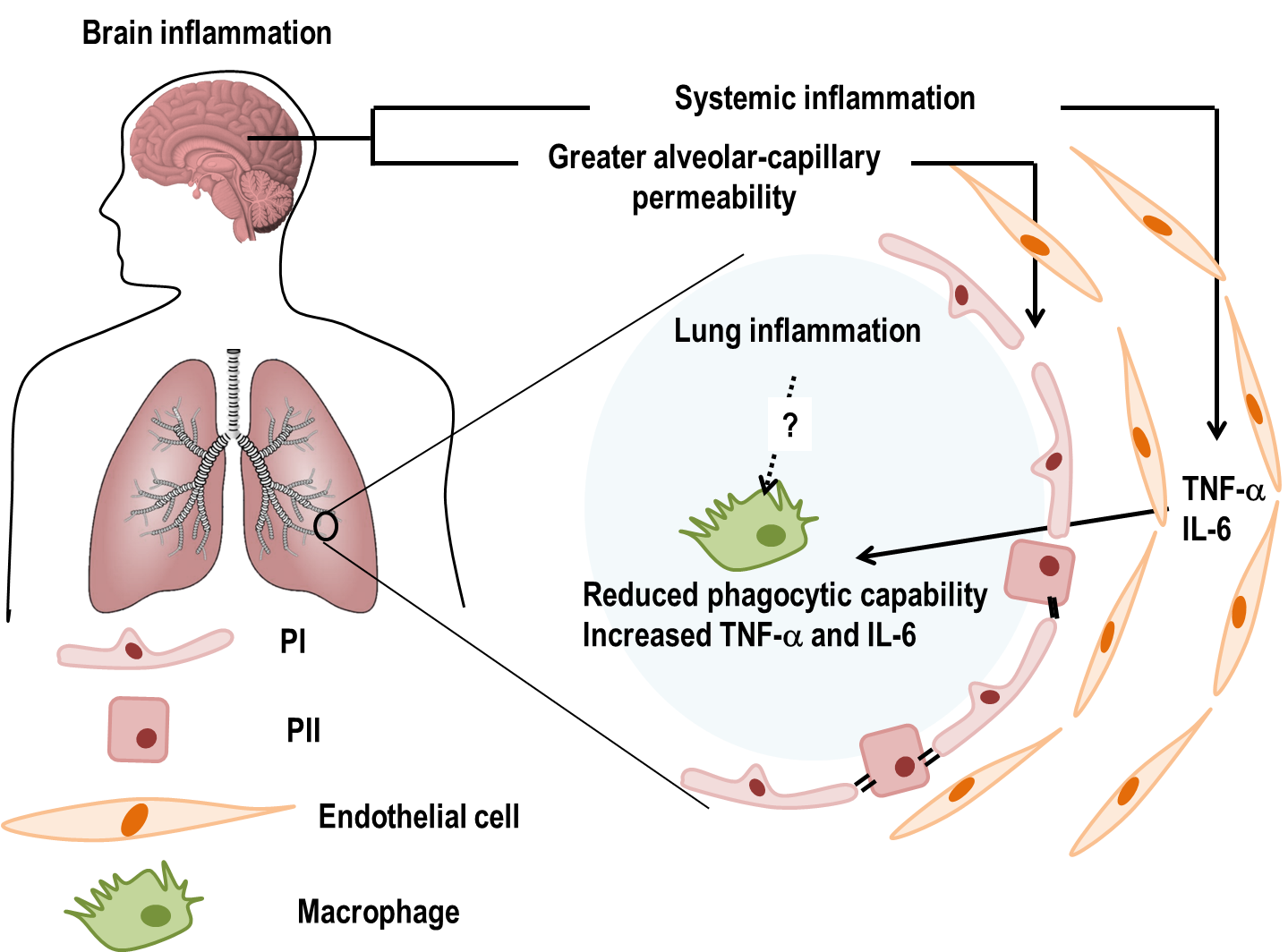


**Figure S9**. Schematic representation of c**rosstalk between the brain and the lung.** Brain damage triggers local and systemic inflammation. The cholinergic anti-inflammatory pathway and the release of glucocorticoids, catecholamines, and damage-associated molecular patterns increase sympathetic activity, leading to greater alveolar–capillary membrane permeability, edema, and inflammation. The release of inflammatory mediators in BALF was not enough to change alveolar macrophage phenotype. Nevertheless, systemic inflammation modified the alveolar macrophage phenotype, reducing alveolar macrophage phagocytic capability and increasing expression of tumor necrosis factor-α and interleukin (IL)-6 mRNA. PI: type I alveolar epithelial cell; PII: type II alveolar epithelial cell.
